# Free-Standing Metal Halide Perovskite Nanowire Arrays with Blue-Green Heterostructures

**DOI:** 10.1021/acs.nanolett.2c00137

**Published:** 2022-03-24

**Authors:** Zhaojun Zhang, Nils Lamers, Chen Sun, Crispin Hetherington, Ivan G. Scheblykin, Jesper Wallentin

**Affiliations:** † Synchrotron Radiation Research and NanoLund, Department of Physics, Lund University, Box 124, Lund 22100, Sweden; ‡ Chemical Physics and NanoLund, Department of Chemistry, Lund University, Box 124, Lund 22100, Sweden; § Centre for Analysis and Synthesis and NanoLund, Department of Chemistry, Lund University, Box 124, Lund 22100, Sweden

**Keywords:** free-standing nanowires arrays, metal halide perovskite, vapor anion exchange, nanowire heterostructure arrays

## Abstract

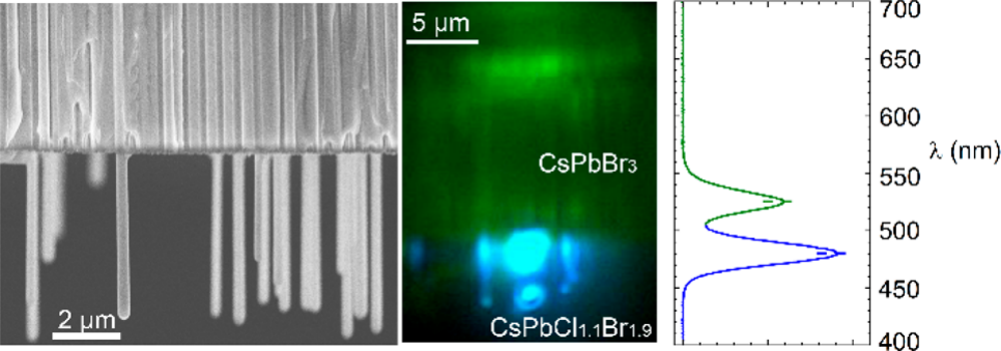

Vertically aligned metal halide perovskite (MHP) nanowires are promising for various optoelectronic applications, which can be further enhanced by heterostructures. However, present methods to obtain free-standing vertically aligned MHP nanowire arrays and heterostructures lack the scalability needed for applications. We use a low-temperature solution process to prepare free-standing vertically aligned green-emitting CsPbBr_3_ nanowires from anodized aluminum oxide templates. The length is controlled from 1 to 20 μm by the precursor amount. The nanowires are single-crystalline and exhibit excellent photoluminescence, clear light guiding and high photoconductivity with a responsivity of 1.9 A/W. We demonstrate blue-green heterostructured nanowire arrays by converting the free-standing part of the nanowires to CsPbCl_1.1_Br_1.9_ in an anion exchange process. Our results demonstrate a scalable, self-aligned, and lithography-free approach to achieve high quality free-standing MHP nanowires arrays and heterostructures, offering new possibilities for optoelectronic applications.

## Introduction

Metal halide perovskite (MHP) nanowire arrays have shown excellent performance in optoelectronic applications such as lasers, LEDs, solar cells, and photodetection.^1−3^ Vertically aligned nanowire arrays offer several advantageous properties, such as enhanced optical absorption and emission along the direction orthogonal to the substrate, which is essential for solar cells and light-emitting diodes,^4−6^ as well as technical advantages including higher nanowire density and scalable device processing. For example, free-standing vertically aligned nanowires of Si, ZnO, GaAs, and InP have achieved excellent performance in various optoelectronic fields.^7−11^ However, the growth of free-standing vertically aligned MHP nanowire arrays is difficult. Compared with the numerous studies on growth of horizontal MHP nanowire arrays,^2,12−17^ there are only a few reports of vertically aligned nanowire arrays, and those mostly concern nanowires physically confined inside various templates such as anodized aluminum oxide (AAO).^18−22^ A few studies have reported free-standing MHP nanowires by solution extrusion^23^ and vapor–liquid–solid^24^ methods, but the alignment of the obtained NWs was not satisfactory. The solution extrusion method works only when the precursor can form an intermediate phase,^23^ while the vapor–liquid–solid growth needs the assistance of a metal catalyst and high temperature.^24^ Therefore, a more general low temperature, catalyst-free method to grow free-standing vertically aligned MHP nanowire arrays is still needed.

Heterostructures are essential for electronic and optoelectronic devices, thanks to their ability to control the electronic band structure, and the ability to create MHP nanowire heterostructures would be highly desirable due to their potential applications such as multicolor displays, self-powered photodetectors, and large-scale electronic circuits.^12−15^ However, the epitaxial growth used to create heterostructures in traditional semiconductors is very difficult in MHPs, since the liquid precursor solution of the new layer tends to dissolve previous layers. Instead, several groups have investigated the halide or anion exchange method, where the halide atoms are replaced after crystal growth.^25,26^ Single nanowire or horizontal nanowire arrays with heterojunctions have been made by solid diffusion,^27^ solution,^28,29^ or vapor anion exchange.^30^ Electron beam lithography was usually required to selectively expose parts of the nanowires before the anion exchange,^28,29^ but such an approach is not scalable for large-area devices.

In this work, we demonstrate free-standing vertically aligned CsPbBr_3_ nanowire arrays that are grown from AAO nanopore substrates. First, the AAO nanopores are filled with CsPbBr_3_ nanowires to act as the substrate for the subsequent epitaxy. In the second phase, free-standing micrometer length nanowires grow from the original nanowires that had formed inside the AAO nanopores. Structural and photoluminescence characterization results show that the nanowires are single crystals with high quality. Using a Cl_2_ vapor phase anion exchange, the free-standing part of the nanowires is converted into CsPbCl_1.1_Br_1.9_ with strong blue photoluminescence, creating an array of millions of aligned nanowires with CsPbBr_3_–CsPbCl_1.1_Br_1.9_ heterojunctions in a single step. Our results demonstrate an efficient method for growing vertically aligned free-standing perovskite nanowires and heterojunction arrays, which will help promote the development of one-dimensional MHP materials and their photonic and optoelectronic applications.

## Results and Discussion

**Figure 1:**
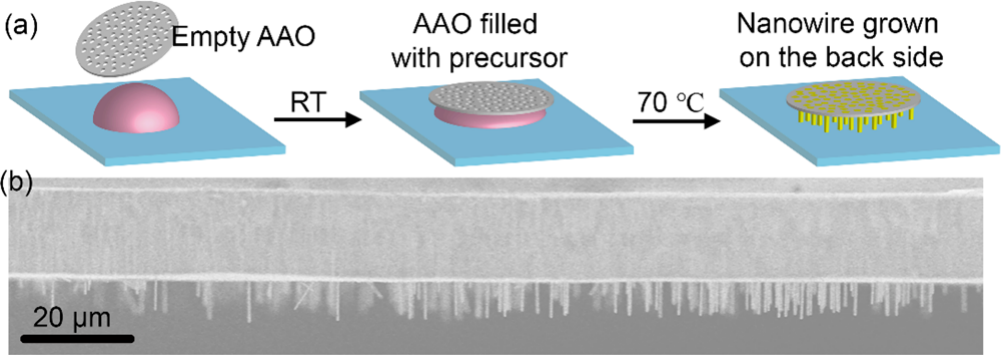
(a) Schematic diagram of the growth process. (b) Cross-sectional SEM images of the as-grown samples with free-standing nanowires on the back side of the AAO template.

A schematic diagram of the growth process is shown in Figure 1a. First, the CsPbBr_3_ precursor (0.3 M CsBr and PbBr_2_ in DMSO) solution was dropped on a cleaned and smooth substrate. Then AAO membrane was put on top of the precursor droplet on the substrate and was infiltrated with precursor solution by capillary forces after 1–2 min. Since the precursor amount is larger than the volume of the AAO pores, extra precursor will remain under the bottom surface of AAO. Then, the whole sample was transferred to a hot plate and heated at 70 °C for 12 h. At the end, vertically aligned free-standing CsPbBr_3_ nanowires grew on the back side of the AAO template, as shown in the cross-sectional SEM image in Figure 1b.

**Figure 2:**
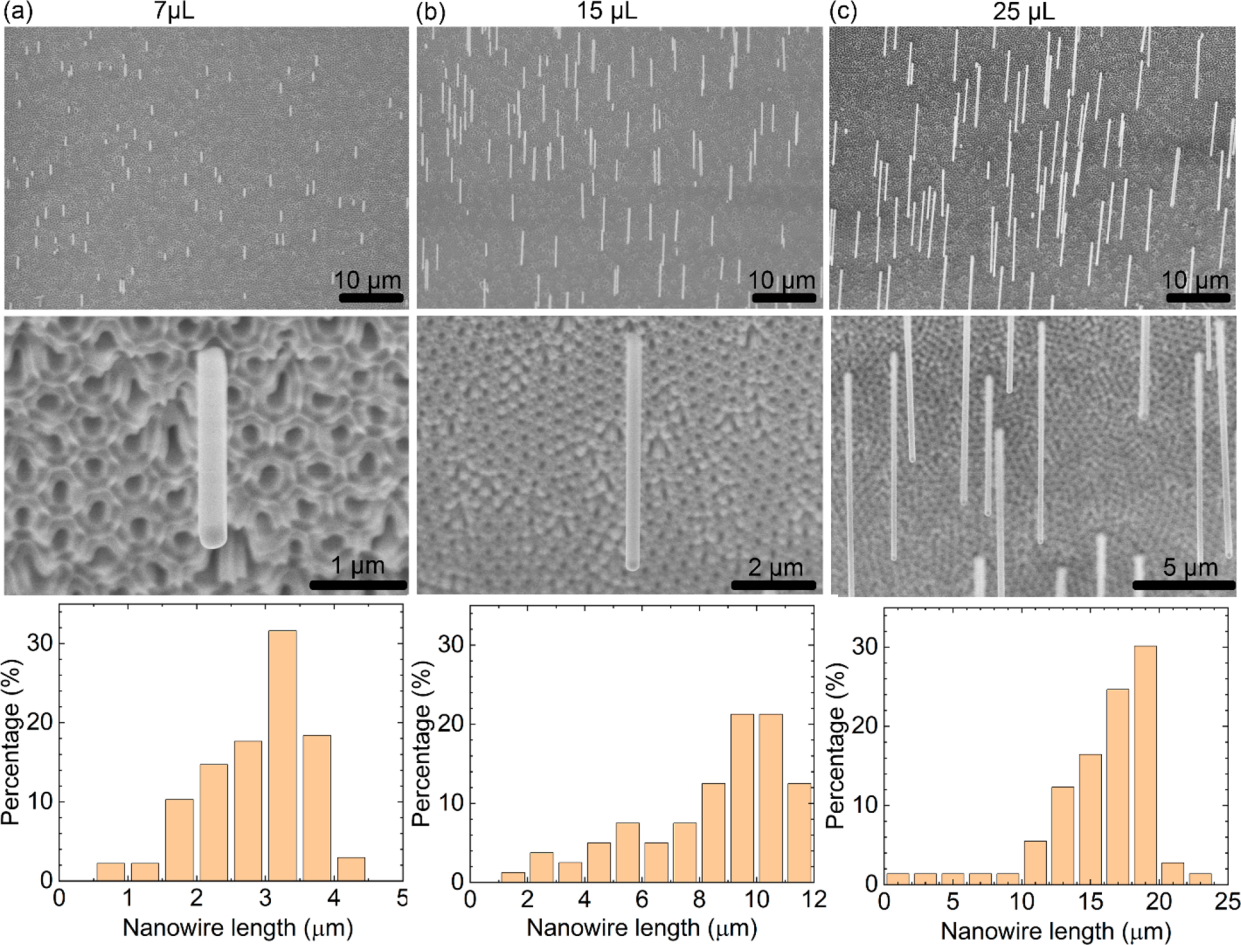
Control of nanowire length. SEM images and statistical distribution of the length of the free-standing nanowire arrays, grown by using (a) 7 μL, (b) 15 μL, and (c) 25 μL precursor solution. The sample stage is tilted by by 30°, that is, the nanowires are twice as long as they appear in the SEM.

By using different amount of precursor, the obtained nanowire length could be controlled. We show SEM of the nanowires grown using 7, 15, and 25 μL precursor in Figure 2, where the sample was tilted 30°. All of the nanowires are vertically aligned with the same growth direction. With increasing amount of precursor, the average nanowire lengths increased to (a) 3.2 ± 1, (b) 10 ± 2, and (c) 18 ± 3 μm, and as shown in Figure S1 the nanowire length increased linearly with increasing precursor amount. The intercept at (3.3 μL, 0 μm) indicates that there will be no free-standing nanowires grown when the precursor amount is less than 3.3 μL, since this amount is needed for the growth of the nanowires inside the AAO.

In contrast, the measured nanowire density was approximately constant at (a) 14000/mm^2^, (b) 16000/mm^2^, and (c) 14000/mm^2^, respectively, meaning that the number of free-standing nanowires do not increase with time. The SEM shows that the distance between nanowires is relatively homogeneous without any nanowires very close to each other. Presumably, it is the in-pore nanowires that first reach the surface that form free-standing nanowires. Our interpretation is that once a free-standing nanowire has formed, it consumes the precursor within a certain collection area and prevents further nanowires to form nearby.^31^

As the high-resolution SEM images show, the free-standing nanowires have a square cross-section. The nanowire diameter is defined by the pore diameter and does not change for different precursor amounts. Even for the 20 μm long nanowires, we did not observe any diameter variation along the nanowire axis, meaning that the growth rate of the nanowire side facets is much smaller than the axial one. These results demonstrate an excellent uniformity and control of the nanowire array growth. We tried different AAO pore diameters in a range from 10 to 350 nm but only obtained free-standing nanowires in the range from 200 to 350 nm. However, we did manage to grow nanowires inside the pores for all diameters,^21,22^ and it is quite possible that thinner free-standing nanowires could be obtained using optimized growth conditions.

**Figure 3:**
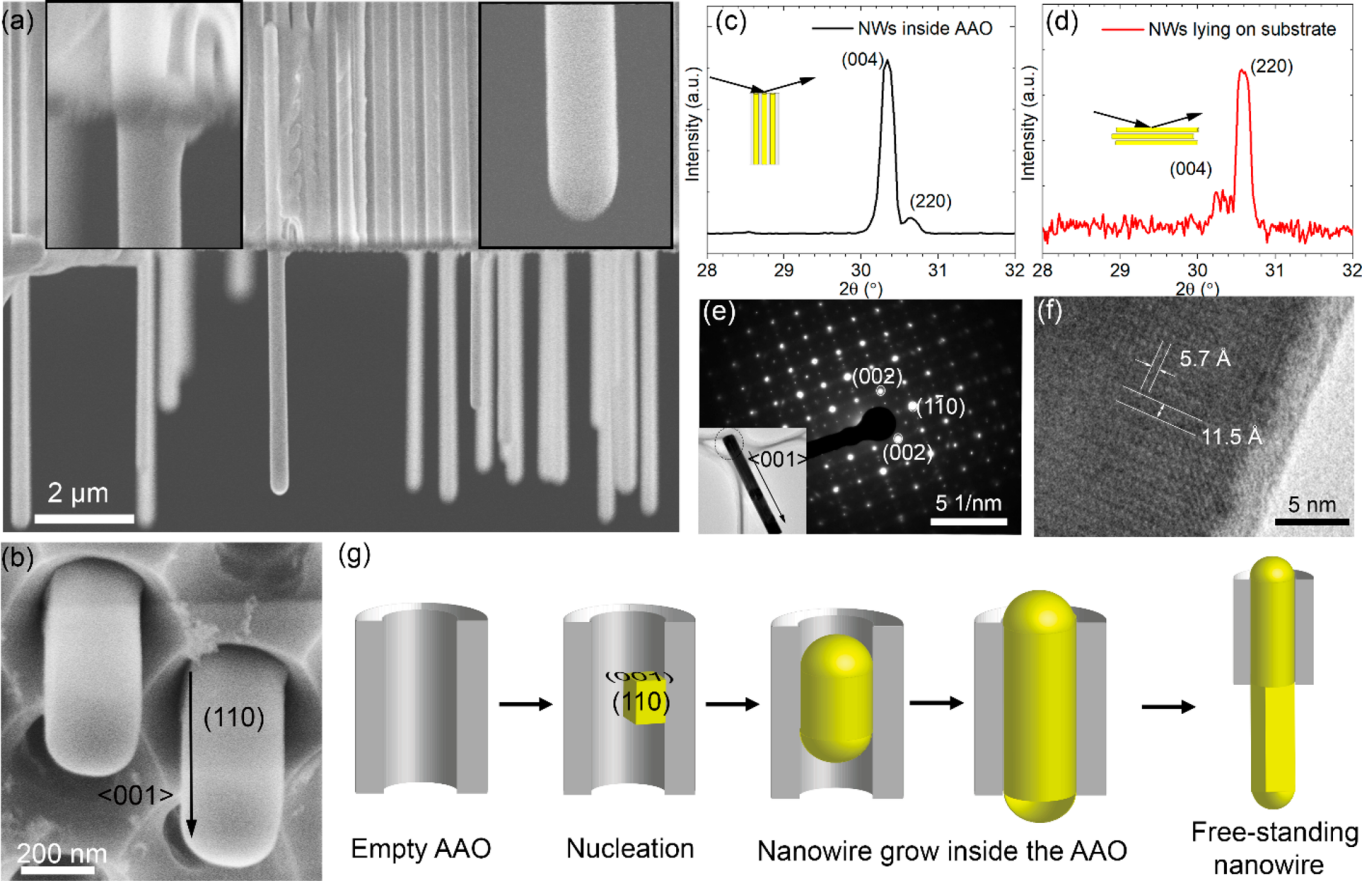
Structural characterization (a) Cross-sectional SEM images of free-standing nanowires grown from the AAO. (b) Tilted top view of the free-standing nanowires from the AAO pores. XRD patterns of nanowires inside AAO (c) and free-standing nanowires transferred onto another substrate (d). The insets show the diffraction geometries. (e) SAED pattern and low-magnification TEM image. (f) HRTEM image of a single CsPbBr_3_ nanowire. (g) Schematic diagram of the growth mechanism of the free-standing nanowires from the AAO nanopores.

In order to further characterize the nanowires and their relation with the AAO template, we used high magnification cross-sectional SEM images. As shown in Figure 3a, the free-standing nanowires originate from nanowires inside the AAO pores. The cross-section of the nanowire inside the AAO pores is approximately round, defined by the nanopores (as seen from the top view SEM of the AAO nanopores in Figure S2). However, the free-standing nanowires have distinct surfaces with a square cross-section as seen in Figure 3b. Additionally, as seen from Figure 3a,b, the tips of the nanowires have a hemispherical shape (SEM images showing the tips of other nanowires are shown in Figure S3).

To understand the growth mechanism of the nanowire, we investigated the crystal structure and growth direction of the CsPbBr_3_ nanowires by XRD. We collected XRD patterns of nanowires in the AAO pores as shown in Figure 3c with the scattering vector along the pores. We also transferred free-standing nanowires to a Si substrate and collected their XRD pattern in a specular geometry as shown in Figure 3d. The full range of patterns with 2θ from 10 to 40° is displayed in Figure S4. In the second case, the nanowires were resting on their largest facets but with a random in-plane orientation; since the scattering vector was orthogonal to the substrate, this geometry probed crystal planes parallel to the facets. For the nanowires inside the AAO, the main diffraction peak is the (004) peak of orthorhombic CsPbBr_3_ (*Pnmb*, *a* = 8.207 Å, *b* = 8.255 Å, and *c* = 11.759 Å), which shows that the nanowires have (001) planes orthogonal to their long axis. For nanowires lying on the substrate, in contrast the strongest diffraction peak is consistent with the (220) peak of orthorhombic CsPbBr_3_, demonstrating that the nanowires primarily have (110)-type facets.

We also performed TEM investigations to further corroborate these results. Figure 3e displays a low-magnification TEM image and an SAED pattern of a single nanowire, while Figure 3f shows a high-resolution TEM image. The lattice spacings of 11.5 and 5.7 Å correspond to the (001) and (110) crystal planes of orthorhombic CsPbBr_3_, and agree with the SAED pattern and XRD results. From the XRD and TEM analysis, we conclude that the nanowires are single crystal and grow in the orthorhombic phase along the ⟨001⟩ direction. The element mapping of a single nanowire (Figure S5 and Table S1) revealed a uniform elemental distribution of Cs/Pb/Br close to 1:1:3. This result strongly suggests that all nanowires including their tips were pure phase CsPbBr_3_.

From the above analysis, we find that the NWs have preferred (110) side facets and grow along the ⟨001⟩ direction. According to the Gibbs–Curie–Wulff theorem, the crystal grows into the shape with minimum total surface energy.^33^ Therefore, the observation that the (110)-type facets are the largest shows that they have the lowest surface energy in our growth conditions, lower than the (001). In contrast, previous DFT calculations indicate that for orthorhombic CsPbBr_3_, (110) has almost the same surface energy as the (001) interface.^34^ However, the relevant interfacial energies in our growth system are the solid–liquid interfaces between CsPbBr_3_ and the precursor solution at the growth temperature, not solid-vacuum as in the theoretical calculations. Chen et al. also reported an increased difference in surface energies between (110) and (001) planes for growing CsPbBr_3_ nanowires.^17^

According to the above analysis, the proposed growth mechanism is shown schematically in Figure 3g: The nanowires first nucleate inside the pores, since the AAO nanopores have ample surface area for heterogeneous nucleation.^21^ The nanowires preferentially nucleate with the (001) planes orthogonal to the pores, that is, with the (110) side facets parallel to the AAO pore walls, presumably because (110) has the lowest solid–solid interfacial energy. Once the nanowires reach the top of the pores, the (001) top facets become substrates for subsequent growth. The remaining supersaturated solution supplies material for continuous growth without the need for overcoming barriers for nucleation at new sites. The (001) top facet has a higher solid–liquid interfacial energy than the (110)-type side facets, which promotes nanowire growth along the ⟨001⟩ direction. In the end, free-standing CsPbBr_3_ nanowire arrays are produced.

Nanowires were found to also grow out of the pores from the top side of the AAO pores, but their morphology was quite different as shown in Figure S6. The nanowires grown from the top side of the AAO were often bent and had a much higher density than the growth from the back side. However, from our characterization of hundreds of samples, we found that the growth from the top side of the AAO was much less controlled than from the back side. This is possibly because the precursor on the top surface is directly exposed to air, and the interface between the air and the precursor may cause nucleation and growth of thin film before the nanowires inside the AAO grow into free-standing nanowires. Figure S7 shows a typical cross-sectional SEM image of a sample with a thin film on the top surface.

**Figure 4:**
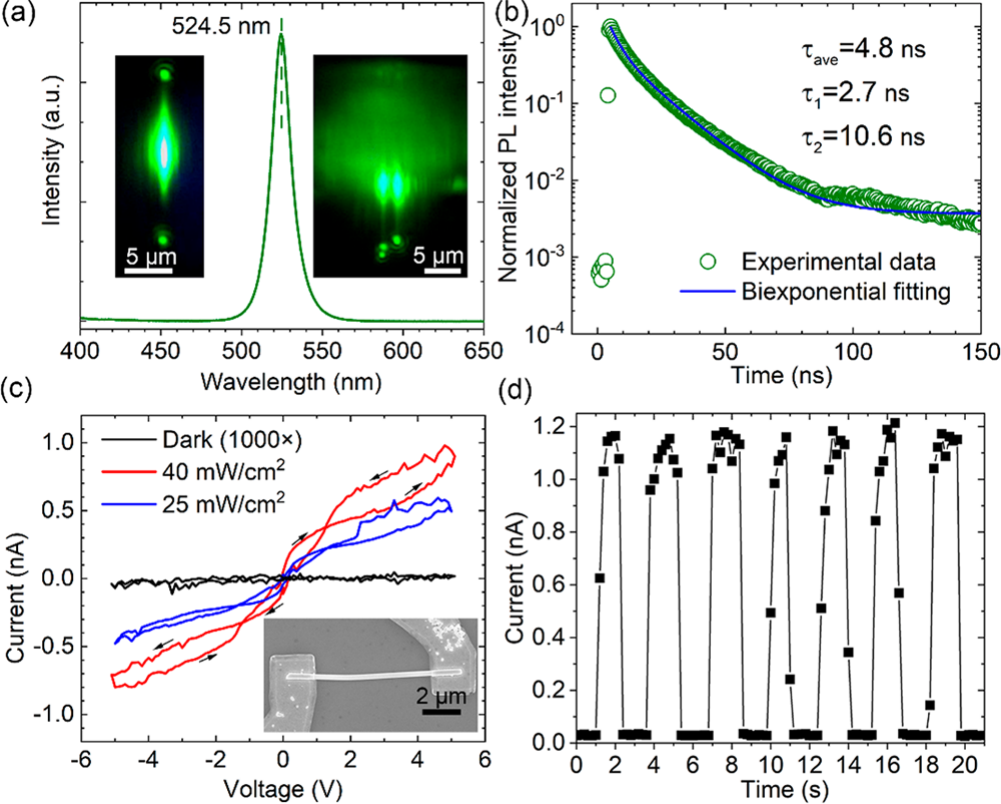
Optical and electrical properties. (a) Photoluminescence spectrum of a single nanowire. The left inset shows an optical image of the transferred single nanowire, while the right inset shows as-grown free-standing nanowires in a cross-sectional view under the excitation of a focused 378 nm laser, aligned onto the center of the images. (b) Time-resolved photoluminescence decay profile of the nanowires. (c) *I*−*V* curve of a single nanowire transistor in dark condition and under illumination of 25 and 40 mW/cm^2^ 405 nm light. (The voltage scan is from 0 to −5 V, then to +5 V and finally back to 0 V in steps of 0.1 V.) The dark current is shown multiplied by a factor of 1000 for clarity. The inset shows a SEM image of the device. (d) Current generated by the device under periodical illumination of 40 mW/cm^2^ 405 nm light at an electrical bias of 5 V.

Good optical properties are essential for most applications of MHPs, and we therefore investigated the photoluminescence of the nanowire (Figure 4a). The single nanowire has green emission centered at 524.6 nm, and a peak width of 14.7 nm. We measured the photoluminescence spectra from 20 nanowires, which showed an emission centering on average at 523.4 ± 2.6 nm with a fwhm of 15.7 ± 2.2 nm. These values are close to the results from previous studies of CsPbBr_3_ nanowires.^12,16,35−38^ The narrow width and symmetrical shape of the emission peak is an indication of high crystalline quality of the nanowires. The left inset shows an optical image of a transferred single nanowire, where a focused laser excites at the center and light guiding leads to emission at both ends. The right inset demonstrates light guiding in as-grown free-standing nanowires. Figure 4b shows the biexponential decay fitting function, () of the time-resolved photoluminescence decay profile of the free-standing nanowires. The decay has characteristic lifetimes of τ_1_ = 2.7 and τ_2_ = 10.6 ns, and the amplitude-averaged lifetime  The fitting parameters are shown in Table S2. We tentatively attribute the short and long lifetimes to nonradiative and radiative recombination of free carriers, respectively.^39^ Regarding the long-term stability, we have found that the as-grown nanowires still have strong and stable green luminescence after 8 months storage under ambient conditions.

We also made a basic investigation of the photoconductivity of the nanowires. Free-standing CsPbBr_3_ nanowires were transferred to an insulating substrate and connected with metal contacts to create nanowire transistors, using a recently reported electron beam lithography process based on nonpolar solvents.^40^Figure 4c shows the current–voltage curve of the device under a dark condition and under illumination of 25 and 40 mW/cm^2^ 405 nm light. The dark *I*–*V* curve was multiplied by a factor of 1000 for the plot in Figure 4c and shown in original scale in Figure S8. At 5 V bias, the dark current is as low as 50 fA. Under illumination of 405 nm light with power density of 40 mW/cm^2^, the photocurrent is 1.15 nA at 5 V bias. The ratio *I*_light_/*I*_dark_ is up to 2.2 × 10^4^, and the responsivity was calculated as , where *P* is the light power, and *A* is the effective illuminated area of the nanowire. This responsivity is much higher than some previously reported CsPbBr_3_ polycrystalline thin film photodetectors.^41,42^Figure 4d shows the response of the device under periodic light illumination. The response time is faster than the data collection rate of the electronics, 0.2 s. We also performed a long-term *I*−*V* test of the single nanowire transistor, which showed increasing conductivity. This effect can be attributed to ion migration of the CsPbBr_3_ single nanowire device under applied long-term bias.^43^ The conductivity increase can be recovered after storing the device under zero bias for several hours, allowing the ions to diffuse back to their initial distribution. The fast response time and good responsivity of the transistor indicate the high crystalline quality and good electronic properties of the nanowire and indicate the potential applications of free-standing CsPbBr_3_ nanowire arrays in various electronic and optoelectronic fields.

**Figure 5:**
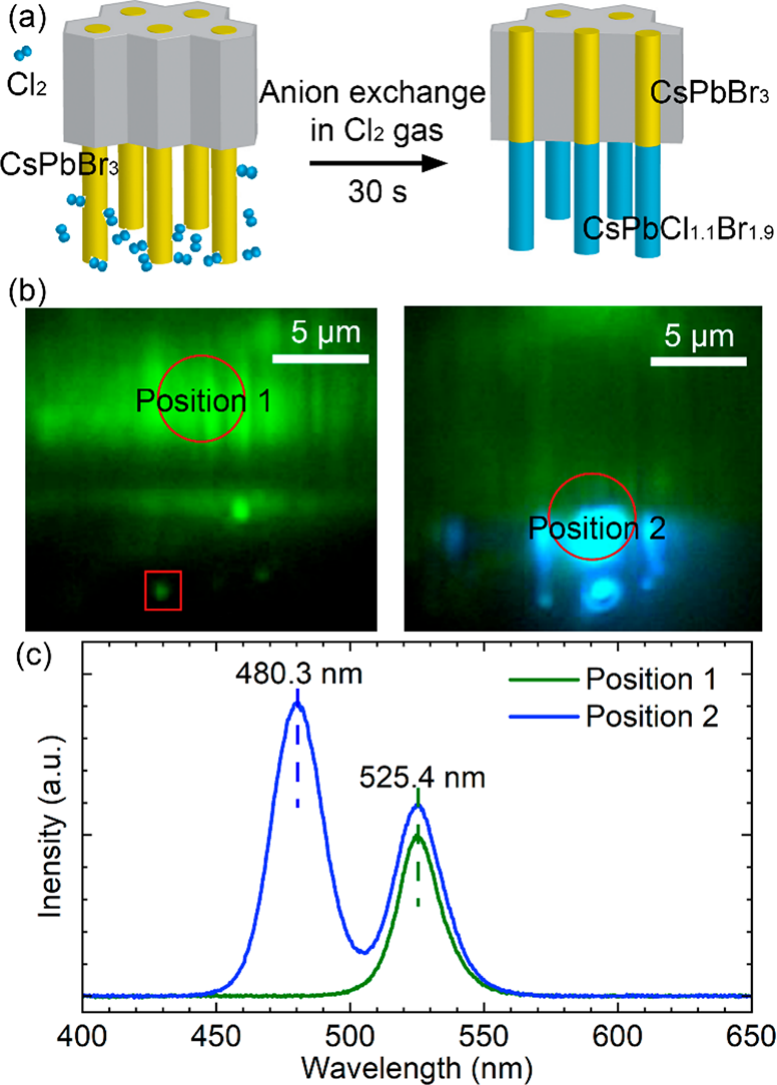
Free-standing nanowire blue-green heterojunction nanowire array (a) Schematic diagram of the anion exchange process. (b) Cross sectional microscopy images of the same heterojunction nanowire array sample under excitation of focused UV laser at different positions. Position 1: laser excited at the AAO part (left); all of the nanowires show green emission. The green spot in the red square indicates that the free-standing nanowire has light guiding of the green light. Position 2: laser excited near the heterojunction (right). The nanowire segments inside the AAO have green emission and the free-standing segments have blue emission. (c) Photoluminescence spectra under excitation of UV laser at the different positions on the heterojunction array, as indicated in (b).

Nanowire heterostructures are highly interesting for applications and basic science, but current methods based on lithography lack scalability.^28,29^ In our case, part of the nanowire is free-standing and part is confined inside the AAO template. We can take advantage of this arrangement as follows: we put the sample in a Cl_2_ gas environment for 30 s to cause a vapor anion exchange process, as shown in Figure 5a. The free-standing nanowires are exposed to the anion exchange process, while the nanowires inside the AAO are protected. Consequently, millions of heterojunction nanowires are fabricated in a single, lithography-free step.

The blue-green emitting heterostructured nanowires array sample were imaged by cross sectional optical microscopy (Figure 5b), where the laser spot excited different positions (red circles). The two images in Figure 5b are from the same region of the same sample, but when the laser excitation is at different positions, the corresponding spectra are totally different as plotted in Figure 5c. For position 1, the laser irradiated the nanowire segments inside the AAO which show green emission centered at 525.4 nm. Note that the free-standing nanowires still provide light guiding of the green light (red square). For position 2, the laser irradiated the interface region between the free-standing nanowire and the nanowire inside the AAO. The light guiding of the blue emission of the free-standing nanowires is clearly seen. Spectrum 2 has two distinct peaks located at 480.3 and 525.4 nm, which are attributed to the free-standing nanowires and the nanowires inside the AAO, respectively. The blue PL emission from the free-standing portion of the nanowires is intense, but the quantum yield cannot directly be compared with the portion of the nanowires inside the AAO since the nanowire density, absorption and emission conditions are very different.

Pure CsPbCl_3_ and CsPbBr_3_ nanowires emit at about 420 nm (2.95 eV)^28^ and 525 nm (2.36 eV), respectively. Using Vegard’s law,^44^ the composition of the part of the nanowire that emitted blue light was estimated to be CsPbCl_1.1_Br_1.9_. We also performed elemental analysis by EDS, displayed in Figure S9, which showed atomic ratios of Br/Cs as 2.3:1 and Cl/Cs as about 0.2:1. The significantly lower Cl concentration from the EDS measurements could be due to Cl loss from the electron beam exposure. Additionally, we also characterized the nanowires after anion exchange by TEM (Figure S10), and the SAED pattern of the nanowires confirmed that the nanowires are still single-crystalline after anion exchange process. The part of the nanowire inside the AAO has a PL emission at 525.4 nm, similar to the pure CsPbBr_3_ nanowires shown in Figure 4a. The EDS measurement in Figure S11 indicates an element ratio of Br/Cs with 3:1 and Cl/Cs of 0.03:1, where the slight trace of Cl signal is within the error margin of the EDS measurement. Thus, we successfully created a free-standing perovskite heterojunction nanowire array by a one-step vapor anion exchange process.

## Conclusions

In summary, this work shows a simple one-step low-temperature method for growing free-standing vertically aligned CsPbBr_3_ nanowires arrays. The obtained nanowires have high single-crystalline quality as confirmed by the structural and optical characterization. A fabricated single nanowire device exhibits excellent photoconductivity, which shows that these nanowires are promising for various optoelectronic applications. Because of the growth behavior of the nanowire, which has a free-standing part and a base part inside the AAO, perovskite heterojunction nanowire arrays were fabricated using a one-step vapor anion exchange process. We believe that our lithography-free and self-aligned method for fabricating free-standing nanowire arrays with heterostructures could be extended to other MHP and non-MHP materials. These results are significant for the future development of MHP nanowire arrays in basic research as well as for optoelectronic and photonic applications.

## Supporting Information Available

Experimental details; the relation of the precursor amount to the averaged nanowire length; top-view SEM images of the empty AAO and AAO with nanowires; cross-sectional SEM image of the free-standing nanowires growing out of AAO nanopores; elemental maps and an XEDS spectrum; the element ratio results table; fitting parameters of the time-resolved PL decay profile; SEM images of the free-standing nanowires from the top surface of AAO template; cross-sectional SEM image of the sample with thin film on the top side of the AAO; *I*–*V* curve of a single CsPbBr_3_ nanowire transistor in the dark condition; the SEM-EDS spectrum, TEM image and SAED pattern of the free-standing nanowire after 30 s in Cl_2_ gas; SEM-EDS spectrum of the nanowire inside the AAO after 30 s in Cl_2_ gas (PDF)
