# Supplementary material for: Free-Standing Metal Halide Perovskite Nanowire Arrays with Blue-Green Heterostructures
Source: Nano Lett. 2022 Mar 24;22(7):2941–7. doi: 10.1021/acs.nanolett.2c00137 (PMC9011394; doi:10.1021/acs.nanolett.2c00137)
Supplement: Supplementary file 1 — nl2c00137_si_001.pdf [file nl2c00137_si_001.pdf]

Supporting information:

Free-standing Metal Halide Perovskite Nanowire Arrays with blue-green Heterostructures

*Zhaojun Zhang[1], Nils Lamers[1], Chen Sun[2], Crispin Hetherington[3], Ivan G. Scheblykin[2], Jesper Wallentin[1]**

[1] Synchrotron Radiation Research and NanoLund, Department of Physics, Lund University, Box 124, Lund, 22100, Sweden.

[2] Chemical Physics and NanoLund, Department of Chemistry, Lund University, Box 124, Lund, 22100, Sweden.

[3] Centre for Analysis and Synthesis and NanoLund, Department of Chemistry, Lund University, Box 124, Lund, 22100, Sweden.

Corresponding author: jesper.wallentin@sljus.lu.se

Experimental Section

Growth of $CsPbBr_3$ nanowire array: The AAO templates were purchased from Topmembranes Technology CO., ltd. The circular AAO templates have a diameter of 13 mm and thickness of 15 μm. The diameter of the nanopores is 200±30 nm. The template was cleaned by ethanol and dried and treated by O_2 plasma to improve the wetting performance of the precursor before use. The precursor solution was made by mixing 638.4 mg CsBr (99.9%) and 1101.0 mg $PbBr_2$ (98%), in 10 mL DMSO (AR, 99.9%) with vigorous stirring for 2 hours until a clear solution was obtained. To test the growth by using different pore diameters AAO, we also used the following different AAO templates. The pore diameter of 10 nm, 20 nm, 30 nm AAO templates all have pitch size of 65 nm. The pore diameter of 110 nm, 250 nm, 300 nm, 350 nm AAO template all have pitch size of 450 nm. And all the AAO templates have a diameter of 13 mm.

Heterojunction by anion exchange: After obtaining the free-standing nanowire array grown from the AAO nanopores, we put the whole sample under Cl_2 gas for 30s. The Cl_2 gas is provided by Advanced Vacuum Apex SLR ICP system with 20 sccm Cl_2 flow and a pressure of 100 mTorr. Before running the process with the sample, we run it once with no sample in order to pre-condition the chamber. It should be noted that the plasma is turned off during the whole process. We only used the Cl_2 flow of the system.

Materials Characterization: XRD measurements were made by using a STOE STADI MP diffractometer in reflection mode with a Cu anode X-ray source (40 kV, 40 mA). The scanning had a step of 0.1° and a speed of 30s/step. The MYTHEN 1K detector collect data with a native data point intervals of 0.015°. The SEM images were obtained on a Hitachi SU8010 electron microscope. EDS spectra were collected using a Zeiss GeminiSEM 500 with a beam incident energy of 10 keV. The TEM was performed in a 300 kV JEM-3000F electron microscope with

an Oxford XEDS system for chemical composition measurement. The photoluminescence spectra were collected with a BWTEM Exemplar plus spectrometer together with a 50× objective and excited with a 375 nm laser in CW mode (OXXIUS). Time resolved photoluminescence is measured using time correlated single photon counting technique. The excitation is provided by a 485 nm pulsed diode laser with a repetition rate range 100Hz-80 MHz, a pulse duration 150-200ps and power up to 3.6 mW. The detector is a PicoQuant PMA hybrid detector with a response time under 130ps.

Device Fabrication and measurements:

First, the free-standing nanowires were transferred onto a substrate by using the common clean cloth used in clean rooms. Then a dual layer consisting of PMMA495 C4 and PMMA950 A5 were spin coated onto the sample. Each layer was spin coated at 3000 rpm for 50 s and baked on a hot plate at 60 °C for 20 min. A Raith 150 EBL system was used for EBL exposure with 20 kV acceleration voltage, 30 μm aperture (roughly 0.3 nA beam current) and 400 $\mu C/cm^{-2}$ exposure dose. The pattern was then developed using a 2:1 mixture of ortho-xylene: hexane for 120 s, followed by a short 3 s dip in pure oxylene to enhance the undercut and clean up the developed pattern. Hexane was used as a rinse solvent. Contacts (20 nm Ti / 200 nm Au) were evaporated using a Temescal e-beam evaporator with the sample at a 30° angle and under constant rotation throughout the process. Lift-off was performed by immersion in 60 °C warm toluene, followed by rinsing in hexane.

The electrical measurements were performed using a Cascade 11000B probe station with a Keithley 4200A-SCS parameter analyzer. A 405 nm green diode laser was used as the light source for the photocurrent measurement. The time-dependent current was measured under a manually controlled light source. As a comparison, excitation with a 653 nm red diode laser did not exhibit any photoconductivity, which confirms that the photoconductivity is caused by the $CsPbBr_3$ nanowire.

All the characterization experiments were made at room temperature.

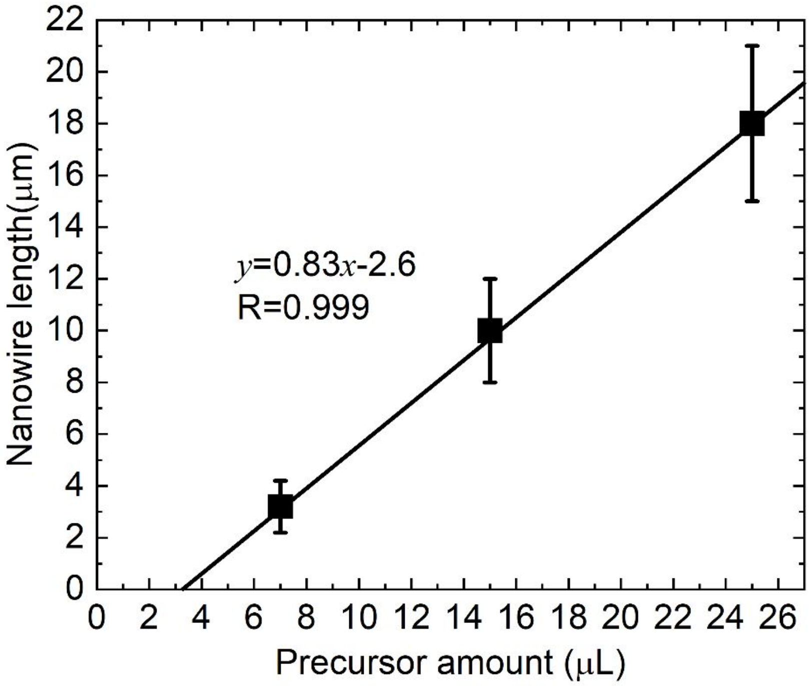
Nanowire length(μm)
Precursor amount (μL)
y=0.83x-2.6
R=0.999
0 2 4 6 8 10 12 14 16 18 20 22 24 26
0 2 4 6 8 10 12 14 16 18 20 22

Figure S1 The variation of the obtained nanowires averaged length grown with different amounts of precursor. The fitting result indicates a linear relation between nanowire length and precursor amount.

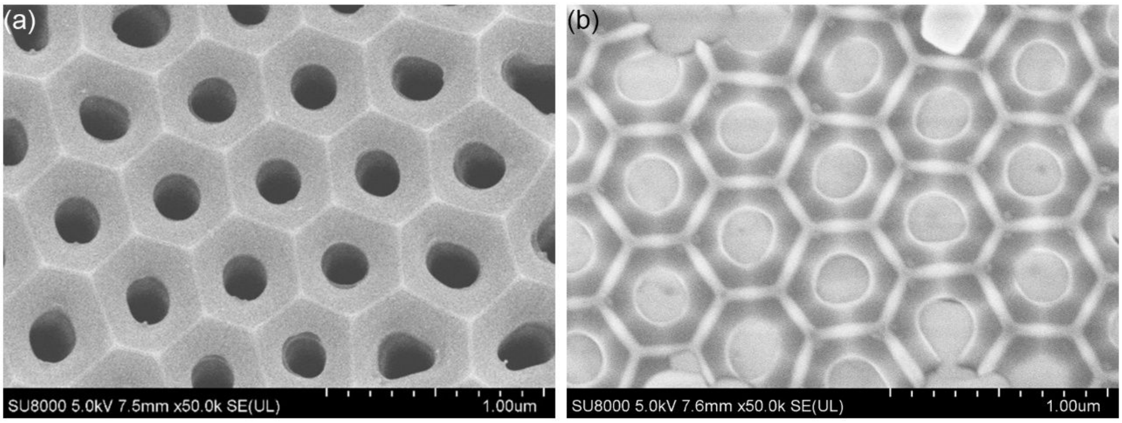

Figure S2 Top view SEM images of the (a) empty AAO nanopores and (b) AAO nanopores with nanowires inside the AAO. The nanopores have a cylindrical shape and pore diameter of about 200 nm.

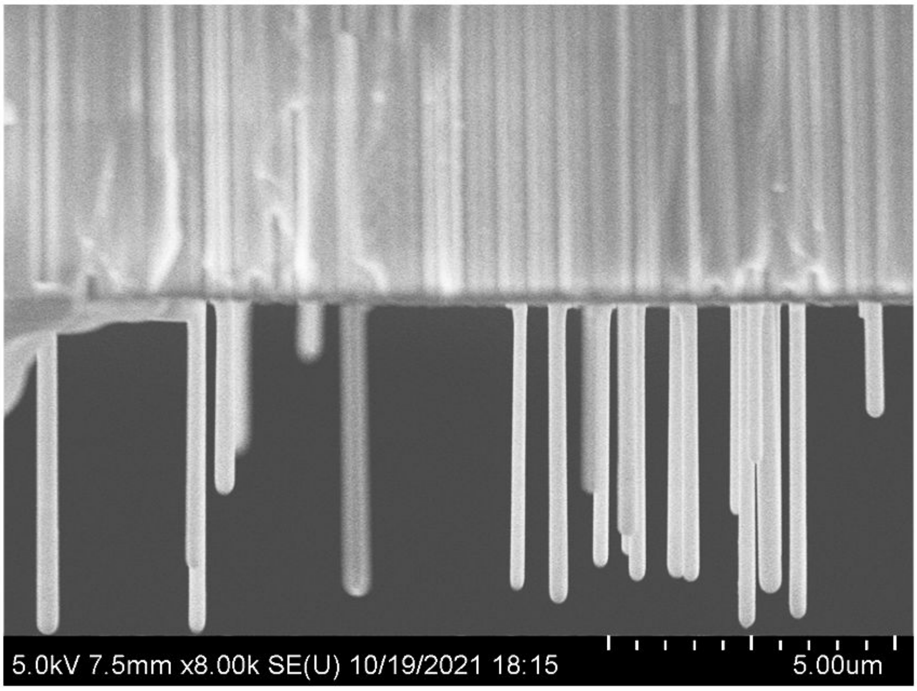

Figure S3 Cross-sectional SEM of the free-standing nanowires growing out of AAO nanopores. This image has a different depth of focus compared to the image in Figure 3a, to achieve sharp images of nanowires at different positions.

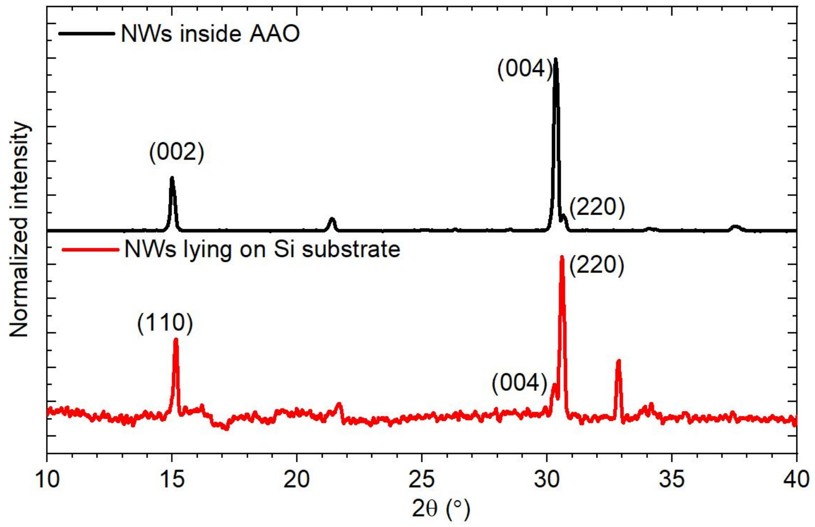

Figure S4: XRD patterns of nanowires inside AAO and of free-standing nanowires transferred to a Si substrate in the full range from 10° to 40°. The peaks at about 33° come from the sample holder for the measurement.

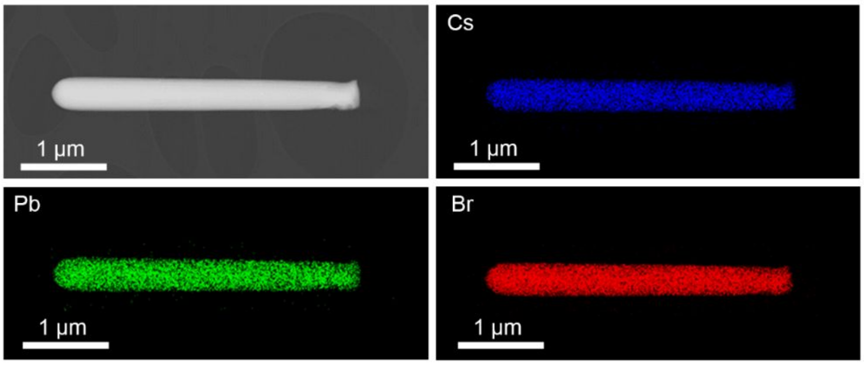

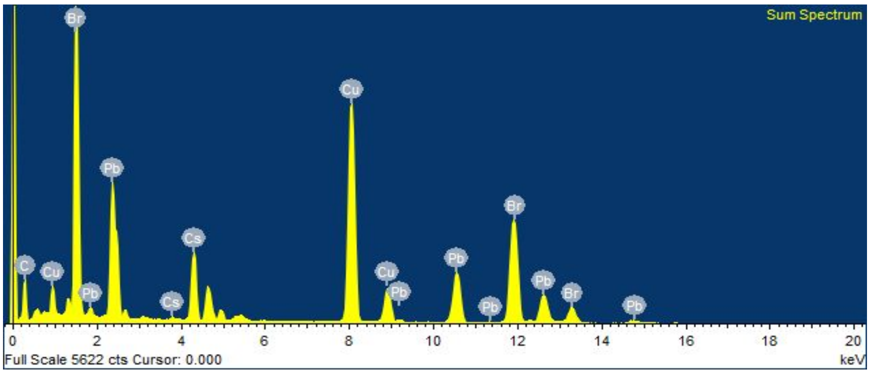

Figure S5: Elemental maps and an XEDS spectrum of a single $CsPbBr_3$ nanowire using.

Table S1 The element ratio results of the single $CsPbBr_3$ shown in Figure S6.

Element	C	Cu	Cs	Pb	Br
Weight%	0.62	28.69	17.01	23.15	30.53
Atomic%	4.61	40.13	11.37	9.93	33.95
Totals	100.00				

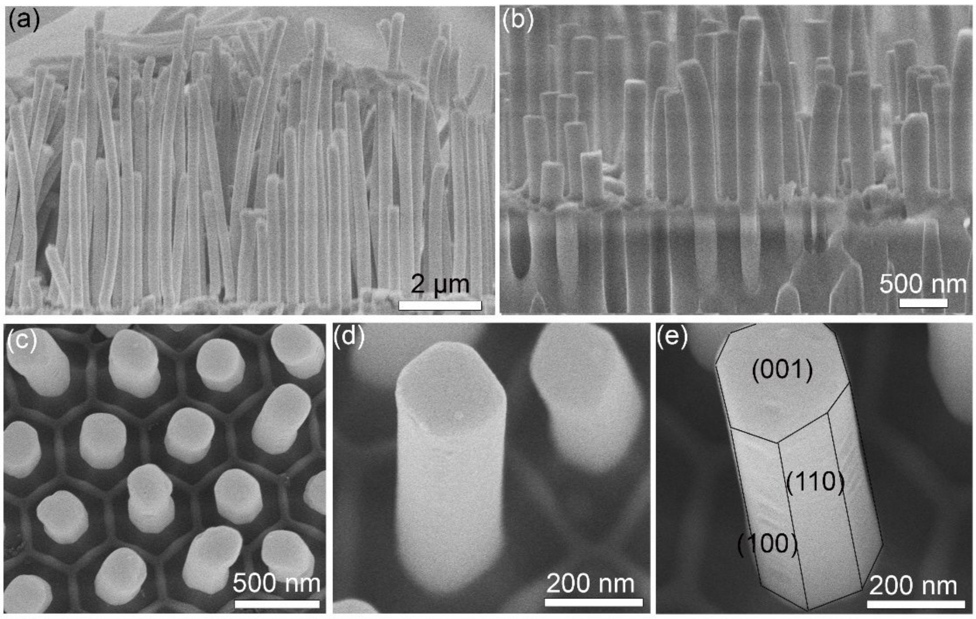

Figure S6 SEM images of the free-standing nanowires from the top surface of AAO template.

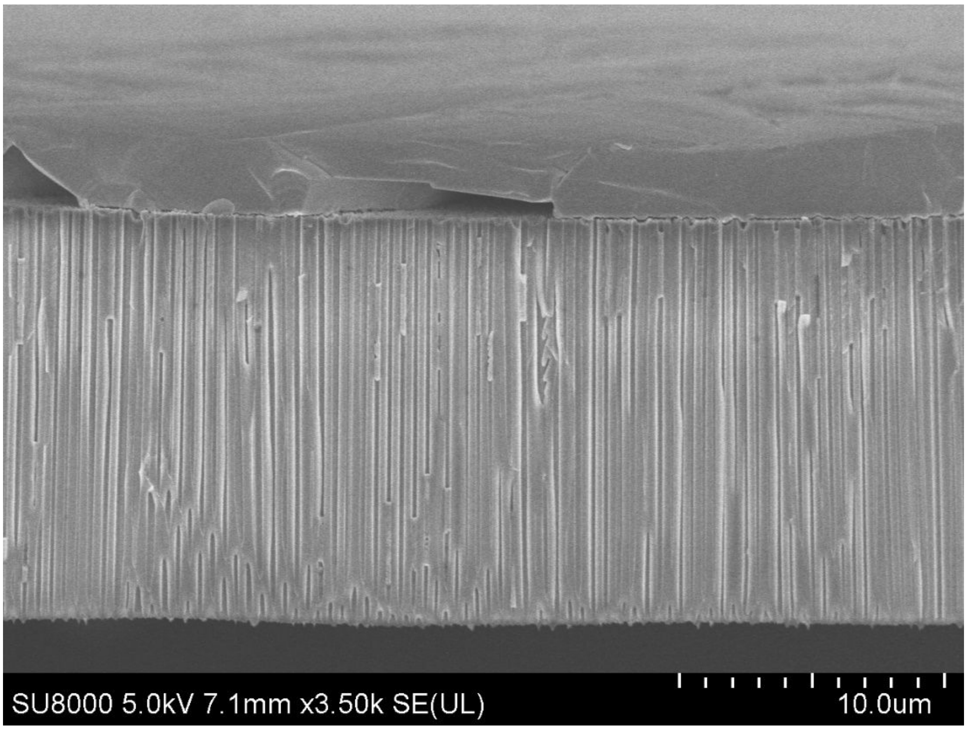

Figure S7 Cross-sectional SEM image of the sample with thin film on the top side of the AAO. There are short nanowires inside the AAO, but the thin film consumed all the precursor before the nanowire reached the top of the nanopores.

Table S2 The fitting parameters of the TRPL decay profile.

Parameter	A_1	A_2	τ_1	τ_2	y_0
Value	1.84	0.67	2.74	10.56	0.0037

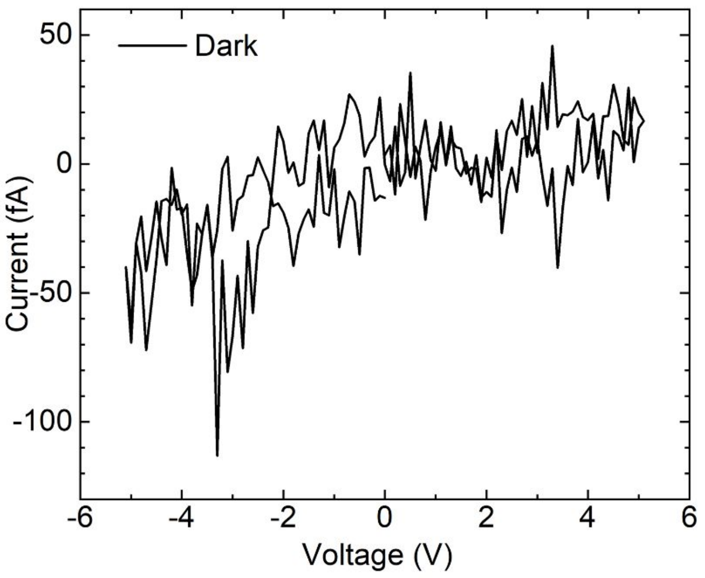

Figure S8 I-V curve of a single $CsPbBr_3$ nanowire transistor in the dark condition.

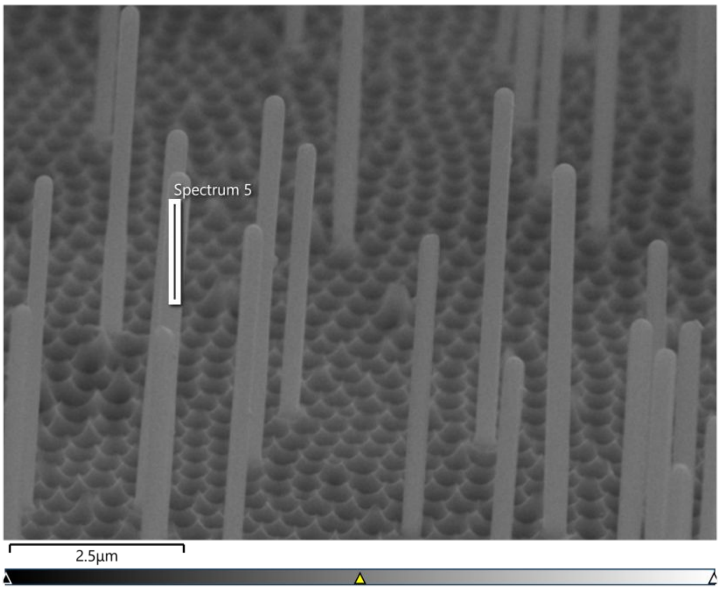

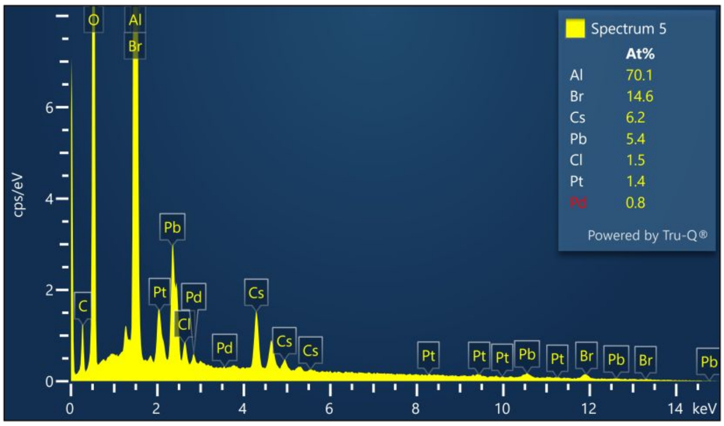

Figure S9 The SEM-EDS spectrum of the free-standing nanowire after 30 s in Cl_2 gas. The square in the SEM image shows the region where the spectrum was acquired.

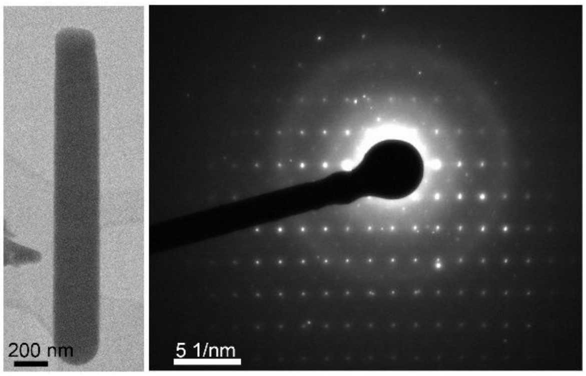

Figure S10 TEM characterizations of the nanowires after anion exchange under Cl_2 gas environment for 30s.

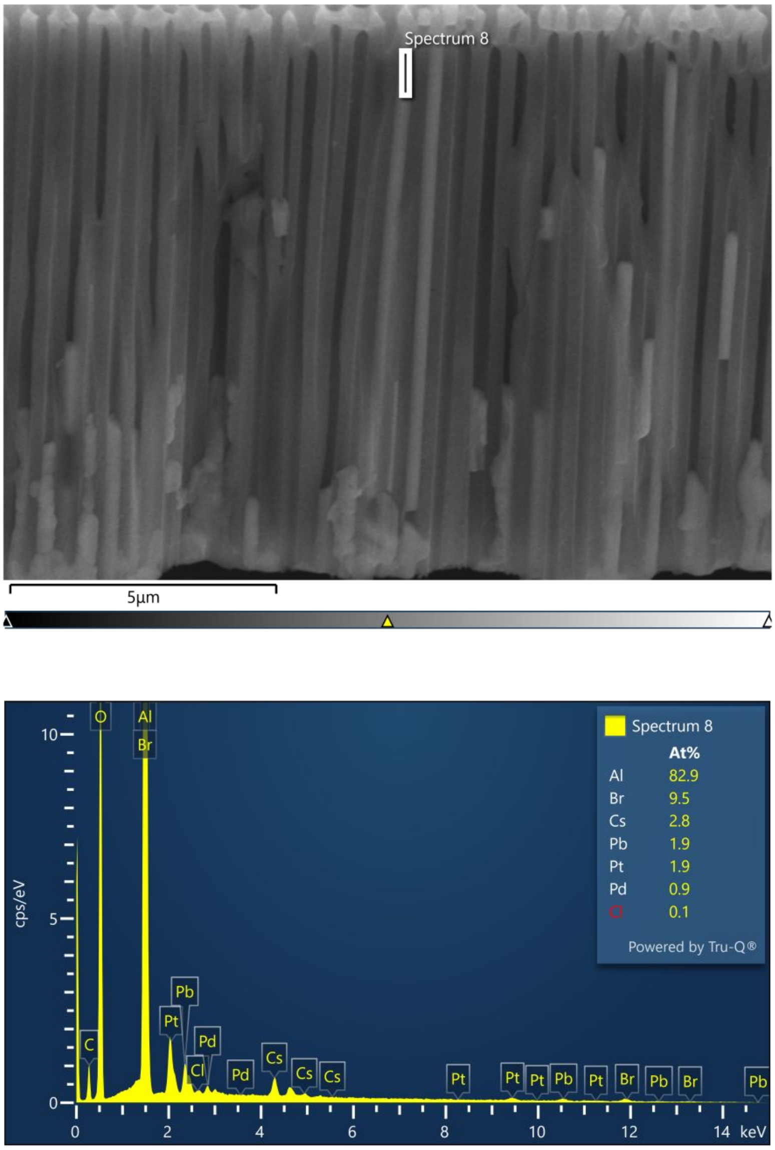

Figure S11 SEM-EDS spectrum of the nanowire inside the AAO after Cl_2 exposure. The square in the SEM image shows the region where the spectrum was acquired.
